# Antiviral activities of multiple antivirals against highly pathogenic avian influenza A H5N1 *in vitro* and in mice

**DOI:** 10.1080/22221751.2026.2645843

**Published:** 2026-03-31

**Authors:** Danlei Liu, Yujing Fan, Ka-Yi Leung, Ruiqi Zhang, Hoi-Yan Lam, Xiaochun Xie, Honglin Chen, Kwok-Hung Chan, Ivan Fan-Ngai Hung

**Affiliations:** a Li Ka Shing Faculty of Medicine, Department of Medicine, University of Hong Kong, Hong Kong, People’s Republic of China; b Li Ka Shing Faculty of Medicine, Department of Microbiology, University of Hong Kong, Hong Kong, People’s Republic of China; c Li Ka Shing Faculty of Medicine, State Key Laboratory for Emerging Infectious Diseases, University of Hong Kong, Hong Kong, People’s Republic of China; d Li Ka Shing Faculty of Medicine, Carol Yu Centre for Infection, University of Hong Kong, Hong Kong, People’s Republic of China; e Centre for Virology, Vaccinology and Therapeutics, Hong Kong Science and Technology Park, Hong Kong Special Administrative Region, People’s Republic of China

**Keywords:** Highly pathogenic avian influenza, H5N1, dairy cow, mouse model, antiviral treatment

## Abstract

In 2024, a bovine H5N1 strain was first isolated from dairy cows in Texas and confirmed to transmit cross-species to humans. Therefore, research on treatments for human infection should be accelerated. In our study, the antiviral effects of baloxavir acid (BXA), oseltamivir carboxylate (OSC), EIDD-1931 (NHC), and ribavirin (RBV) against five H5N1 strains were evaluated *in vitro*. Cell viability and viral replication were measured to assess the antiviral effects. The results showed that the EC_50_ of BXA treatment was the lowest. The BXA/NHC and BXA/OSC combination treatments showed more potent inhibitory effects than each monotherapy. The 15 mg/kg baloxavir marboxil (BXM) / 125 mg/kg molnupiravir (MNP) and the 15 mg/kg BXM / 10 mg/kg oseltamivir phosphate (OSP) were tested in BALB/c mice. The mice were inoculated with 10 times the 50% mouse lethal dose (10 MLD_50_) of bovine H5N1 virus. Treatments began 1-day post-infection (1 dpi) and were administered orally twice daily for 5 or 7 days. Changes in body weight, clinical signs, and survival were monitored; lung and brain tissues were collected for virological, immunological, and histological analyses. Most mice died from severe neurological symptoms. Compared with the 5-day treatment, the 7-day treatment effectively inhibited viral replication and increased survival rates to 50% in BXM, BXM/MNP, and BXM/OSP treatments. Mice treated with BXM/MNP or BXM/OSP combination therapy showed lower viral yields in the lungs than those treated with BXM alone. The results provide a reference for human treatment, and extending the 7-day combination treatment should be considered.

## Introduction

The highly pathogenic avian influenza (HPAI) H5N1 virus poses a significant risk of transmission to humans [1]. The circulating H5N1 viruses in birds, poultry, and many mammals, including clades 2.3.4.4b, 2.3.2.1a, and 2.3.2.1e, have led to human infections [2,3]. In early 2024, the H5N1 virus was first detected in dairy cows in Texas, United States, with high levels detected in raw milk, resulting in a multistate outbreak among cows across the U.S [4]. Since 2024, the virus has been most widespread in the U.S., with 71 cases of cross-species transmission to humans, including 41 cases linked to cattle [5].

The origin of the H5N1 clade 2.3.4.4b virus is a novel reassortment between the H5N8 clade 2.3.4.4b and other avian influenza virus subtypes [6]. The H5N1 viruses isolated from the dairy cows belong to clade 2.3.4.4b, genotypes B3.13 and D1.1. The B3.13 genotype emerged in 2023 through reassortment between Eurasian wild-bird H5N1 lineages and non-H5N1 wild-bird lineages from America [7,8]. The D1.1 genotype was the dominant strain circulating in wild birds in North America from 2024 to 2025. Its detection in dairy cows in early 2025 was the second known case of transmission from wild birds to the dairy cows [9]. The HPAI clade 2.3.4.4b bovine H5N1 virus can bind to human α2,6-linked and avian α2,3-linked sialic acid (SA) receptors, which contribute to species transmission diversity [10]. Mutations at certain positions in hemagglutinin (HA), polymerase basic protein 2 (PB2), matrix protein (MP), and nucleoprotein (NP) of the bovine H5N1 strain have been shown to increase pathogenicity and virulence in mammals [11]. Changes in the HA coding region, such as E91 K, S137F, N209 T, and G240R, increase mammalian adaptation, while mutations such as Q154R, Q234 K/R, S336N, P337L, and R77 K increase pathogenicity and virulence [11]. The mutation (T438A/I) in neuraminidase (NA) may confer potential antiviral resistance [11-13]. Variations in the PB2 and non-structural protein (NS) coding regions are also linked to increased virulence and adaptation to mammals [11]. In March 2024, the H5N1 (Clade 2.3.4.4b, genotype B3.13) virus was first detected in sick cows in Texas, and the deaths of wild birds and domestic cats in the affected areas were also reported [4,8]. Domestic cats fed raw colostrum and milk from sick cows tested positive for the H5N1 virus and showed severe neurological symptoms [4]. In the same month, a dairy farm worker in Texas was confirmed to have an H5N1 virus infection, presenting with initial redness and discomfort in his right eye, without fever or respiratory symptoms, and with normal vital signs [7].

Animal contact is considered the primary way that the HPAI H5N1 virus spreads to humans, especially for high-risk jobs like workers handling poultry, dairy, and other livestock [14]. The H5N1 virus is circulating across different regions and reassorting with other avian and mammalian influenza subtypes, which could lead to increased transmissibility [14]. The reassortment may also influence the severity of infection. A previous study showed that reassortment between avian H5N1 and human H3N2 influenza viruses increased the pathogenicity and virulence of hybrid viruses [15]. NA reassortment linked to oseltamivir resistance was identified in an HPAI H5N1 clade 2.3.4.4b virus that circulated in Canadian poultry in 2024 [6]. Therefore, increased viral transmissibility and adaptability in mammals and humans, along with changes in virulence and drug resistance observed in some H5N1 strains, may affect infection severity and require ongoing adjustments to treatment. A novel HPAI H5N1 clade 2.3.4.4b genotype D1.1 virus was discovered in late 2024 in Canada. Its sequence was most similar to a virus found in wild birds [16]. It caused severe illness in a teenager from British Columbia. Her condition quickly worsened, leading to respiratory distress and hemodynamic instability. She received antiviral treatments, including oseltamivir, baloxavir, and amantadine, underwent tracheal intubation, and was placed on venovenous extracorporeal membrane oxygenation (ECMO), continuous renal replacement therapy (CRRT), and plasma exchange. Ultimately, her condition improved, and she recovered [17]. Since October 2024, four hospitalized cases have also been reported in the U.S. Among these cases, two patients were confirmed to have D1.1 infection, where one was fatal [18]. One fatal case of D1.1 infection in a child was also reported in Mexico [19]. Early diagnosis and antiviral treatment may help with recovery. Some critical cases may be associated with delayed antiviral treatment.

Currently, some antiviral drugs are being considered for the treatment of HPAI H5N1 virus infection. The neuraminidase inhibitors (NAIs) and baloxavir marboxil (BXM) have been recommended for the treatment of HPAI H5N1 influenza virus infection in humans [20,21]. However, conducting clinical trials for the treatment of the novel H5N1 strain is challenging, and there remains a need to improve and expand the recommended treatments in preclinical studies. The mechanism of NAI, such as oseltamivir phosphate (OSP), is to inhibit the activity of NA on the virus surface, thereby preventing the release of the influenza virus [22]. BXM, a cap-dependent endonuclease inhibitor, blocks the polymerase acidic (PA) endonuclease during cap-snatching, ultimately inhibiting viral replication [23,24]. Meanwhile, molnupiravir (MNP), as a broad-spectrum antiviral drug, may have anti-influenza activities. MNP is an oral prodrug that is rapidly converted in host plasma to the ribonucleoside analog N-hydroxycytidine (NHC), which is integrated into viral RNA by RNA-dependent RNA polymerase (RdRp), leading to viral misreplication and inhibiting viral replication [25]. Ribavirin (RBV) is a guanosine analog and has broad-spectrum activity against RNA and DNA viruses [26]. RBV has multiple antiviral mechanisms of action, including inhibition of inosine monophosphate dehydrogenase, which ultimately depletes intracellular GTP, inhibition of mRNA capping and viral RdRp, and effects on host cell gene expression, inflammation, and immune regulation [26].

Given the circulating and highly concerning bovine H5N1 strain, we first tested the effects of multiple antivirals mentioned above and compared them to those of previous H5N1 strains *in vitro*. We then further evaluated the efficacy of drugs against bovine H5N1 strain in mice. The goal was to provide a reference for clinical treatment, particularly for critical cases.

## Materials and methods

The method details are provided in the Supplementary Methods.

### Antiviral efficacy *in vitro*

The bovine H5N1 and four earlier H5N1 strains were studied *in vitro*. Antiviral tests were conducted on MDCK cells. The cytotoxicity concentrations of baloxavir acid (BXA), oseltamivir carboxylate (OSC), EIDD-1931 (NHC), and ribavirin (RBV) were determined by the MTT assay. For the antiviral treatment, five H5N1 strains were diluted to 0.0005 MOI for cell inoculation. After 72 h, cell viability was determined using the MTT assay, and supernatants were collected to test virus titer by using TCID_50_. The 50% effective concentration (EC_50_) was calculated from cell viability results. The 50% inhibitory concentration (IC_50_) of OSC was further calculated from the NA inhibition assay. The A/Thailand/MK2/2004 and recombinant A/dairy cow/Texas/24-008749-003/2024 strains were selected for *in vitro* combination treatments. The dual drug treatment involved BXA combined with NHC, OSC, or RBV. Supernatants were collected at 72 h, and the viral titers were determined by the TCID_50_ method.

### Antiviral treatments in mice

Female BALB/c mice (6-8 weeks old) were intranasally inoculated with 10 pfu (approximately 10 MLD_50_) of bovine H5N1 virus in a total volume of 20 μl after being anesthetized. Six treatment groups were established: 125 mg/kg MNP/15 mg/kg BXM and 10 mg/kg OSP/15 mg/kg BXM combination groups, as well as MNP, OSP, BXM, and placebo groups. All treatments were administered orally twice daily (at 8-hour intervals) starting at 1 dpi and continuing for either 5 or 7 days. Six mice per group were sacrificed at 4 dpi for virologic, immunologic, and histopathologic analyses. Each group of 6 mice was monitored for body weight changes, clinical signs, and survival for 14 or 21 days. Any animal losing more than 20% of its body weight or showing signs of severe disease was euthanized. The evaluation criteria are shown in Supplementary Table 1. The study was approved by the Committee on the Use of Live Animals in Teaching and Research at the University of Hong Kong (CULATR No. 24-171).

### Statistical analysis

The results were analyzed using GraphPad Prism software. EC_50_ and IC_50_ values were calculated through nonlinear regression. The Log-rank Mantel–Cox test was used to analyze survival rates. Normally distributed data were analyzed with One-way ANOVA to compare differences among multiple groups, whereas non-normally distributed data were analyzed with Kruskal–Wallis tests. A *p* < 0.05 was considered statistically significant. *p < 0.05, **p < 0.01, *** *p* < 0.001.

## Results

### Antiviral effects of BXA, OSC, NHC, and RBV against H5N1 in vitro

**Figure 1.**
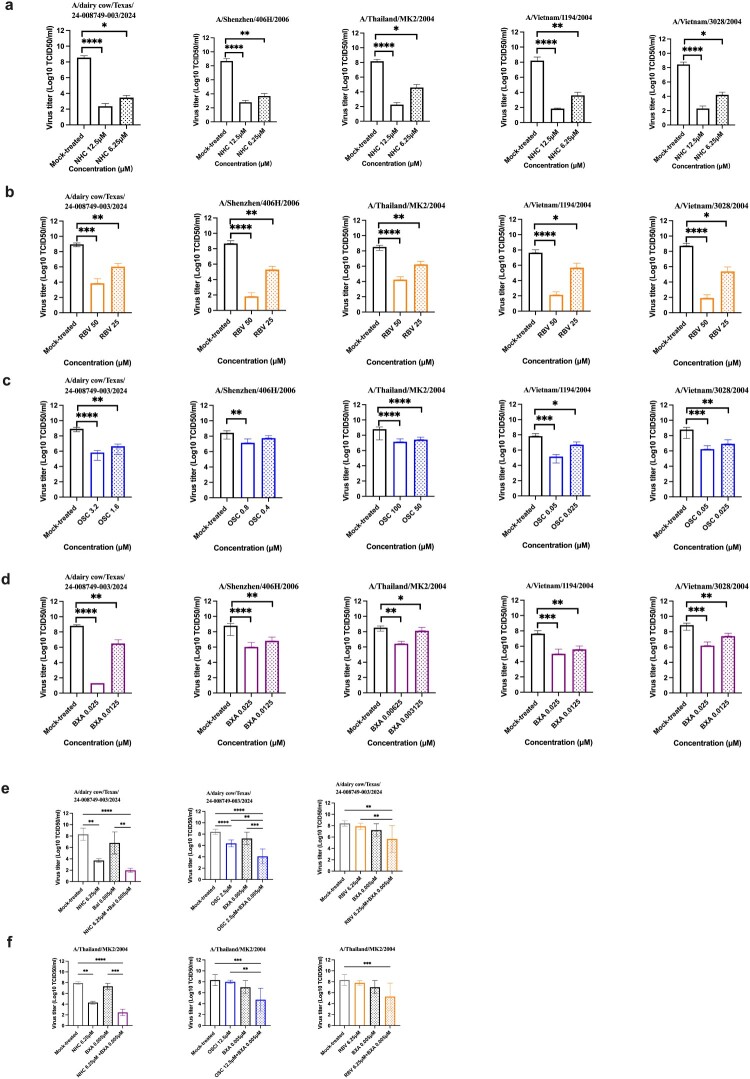
NHC, RBV, OSC, and BXA against H5N1 strains *in vitro*. (a–d) The antiviral effects of NHC, RBV, OSC, and BXA, respectively. (e, f) The combination treatments against A/dairy cattle/Texas/24-008749-003/2024 and A/Thailand/MK2/2004 H5N1 viruses. The virus titer was determined by the TCID_50_ method. The results were obtained from three independent experiments. The results are shown as means ± SD. Statistical analysis was performed using One-way ANOVA. *p < 0.05, **p < 0.01, *** *p* < 0.001.

The efficacy of four antiviral drugs was tested *in vitro*. The highest drug concentrations used in treatment were lower than the CC_50_ and could maintain more than 80% cell viability. BXA had the lowest EC_50_ across all five H5N1 strains. The EC_50_ of OSC against the A/Thailand/MK2/2004 strain (84.10 ± 39.41μM) and the bovine H5N1 strain (4.88 ± 4.00μM) was increased compared to other strains. However, the results of the NA inhibition assay showed that the IC_50_ of OSC against bovine H5N1 strain was 11.06 ± 3.36nM, which was 5–10 times higher than that of the other strains. The EC_50_ of RBV and NHC against the five H5N1 strains was around 40 and 8 μM, respectively. Both BXA and OSC treatments showed higher selectivity indexes (SI) than the other two treatments (Table 1). The EC_50_ and IC_50_ curves for all drugs were shown in Supplementary Figure 1. Supernatants were collected to measure virus titer, and drug concentrations near the EC_50_ significantly reduced the virus titer (*p* < 0.05) (Figure 1(a–d)). Based on monotherapy results, the EC_50_ of OSC against A/Thailand/MK2/2004 and bovine H5N1 viruses was over 50 and 1500 times higher than for other strains, respectively. Therefore, these two strains were selected to evaluate the efficacy of the combination. The results showed that NHC/BXA and OSC/BXA combinations significantly reduced virus yield compared to monotherapies (*p* < 0.05). Compared to the untreated group, 0.005 μM BXA did not show a significant antiviral effect, and the virus titer was reduced by only 1 log10. However, when combined with NHC or OSC, the virus titer could be reduced by 6 log10 and 4 log10, respectively. RBV/BXA also inhibited viral replication, but the virus titer was reduced by only 2 log10 (Figure 1(e, f)).

**Table 1.** Antiviral treatments for different H5N1 strains in vitro.

Virus	Clade	NHC (μM)		RBV (μM)		BXA (μM)		OSC (μM)		OSC (nM)
		EC_50_	SI	EC_50_	SI	EC_50_	SI	EC_50_	SI	IC_50_
A/dairy cattle/Texas/24-008749-003/2024	Clade 2.3.4.4b	8.81 ± 5.78	7.70	41.58 ± 21.90	>48.10	0.011 ± 0.006	1408.18	4.88 ± 4.00	>409.84	11.06 ± 3.36
A/Shenzhen/406H/2006	Clade 2.3.4	8.04 ± 3.79	8.39	36.01 ± 13.05	>55.54	0.012 ± 0.001	1290.83	0.75 ± 0.24	>2666.67	2.78 ± 2.08
A/Thailand/MK2/2004	Clade 1	8.49 ± 1.20	7.95	30.13 ± 8.12	>66.38	0.006 ± 0.001	2581.67	84.10 ± 39.41	>23.78	0.41 ± 0.07
A/Vietnam/1194/2004	Clade 1	8.21 ± 2.88	8.22	39.85 ± 10.87	>50.19	0.015 ± 0.006	1032.67	0.05 ± 0.04	>40000	1.22 ± 1.09
A/Vietnam/3028/2004	Clade 1	8.85 ± 4.21	7.62	38.35 ± 2.36	>52.15	0.019 ± 0.016	815.26	0.04 ± 0.02	>50000	0.54 ± 0.37

Notes: CC_50_: NHC 67.48μM, RBV and OSC >2000μM, BXA 15.49μM. SI: selectivity index, calculated by CC_50_/EC_50_. EC50 was calculated based on cell viability. IC_50_ was calculated based on the NA inhibition assay.

### Efficacy of BXM, OSP, and MNP against H5N1 virus in mice

**Figure 2.**
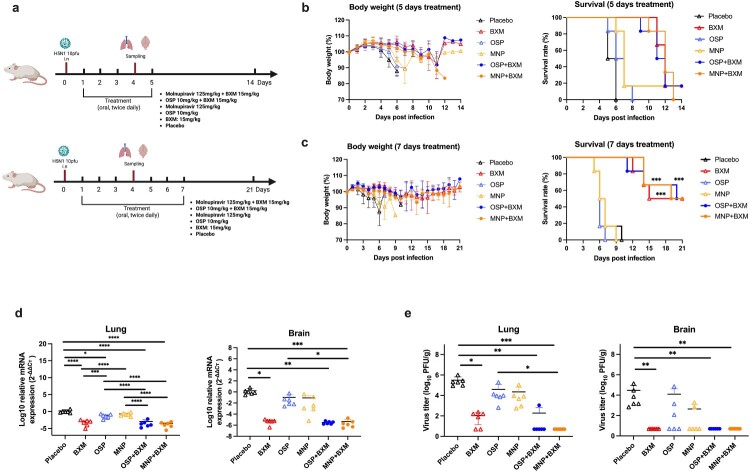
Effects of BXM, OSP, and MNP against the bovine H5N1 strain in mice. (a) The treatment procedure for the animals. The figures were created with BioRender.com. (b, c) Five-day and seven-day treatments, starting at 1 dpi, showing body weight changes, and 14-day survival rates for each group (n = 6). (d, e) Viral replication in the right lungs and right brains of mice at 4 dpi (n = 6). The mRNA levels were measured by RT-qPCR targeting the influenza A M gene, normalized with β-actin, and calculated using the 2^-ΔΔct^ method. The virus titer was determined by plaque assay. The results were shown as means ± SD. Statistical analysis was performed using One-way ANOVA. *p < 0.05, **p < 0.01, *** *p* < 0.001.

The recombinant A/dairy cow/Texas/24-008749-003/2024 H5N1 virus was used in the mouse model. Based on the *in vitro* results, BXM, OSP, and MNP were chosen for testing both single and combined treatments *in vivo*. The 1 MLD_50_ was 1.1 pfu, and the associated body weight loss and survival rates at different viral doses are shown in Supplementary Figure 2. The experimental procedures are outlined in Figure 2(a). The 5-day treatment was initially evaluated. Mice receiving the placebo either died or reached a humane endpoint at 5-6 dpi. The OSP and MNP monotherapies did not significantly prolong survival time (6.5 and 7.0 days, respectively). BXM, MNP/BXM, and OSP/BXM treatments increased survival to 11–12 days. However, the disease continued to progress and caused the deaths of most mice before 14 dpi; only one mouse (16.7%) survived in the BXM, MNP, and OSP/BXM treatment groups, respectively (Table 2). Body weight loss was detectable at 4 dpi, with an initial reduction of < 5%. The clinical scores of the dead mice in each treatment group ranged from 4 to 6 points (Table 2). Nearly all mice reached a humane endpoint or died due to severe, persistent generalized seizures or limb paralysis, with neurological signs accompanied by rapid weight loss. Survival rates did not differ significantly across groups (Figure 2(b)). The treatment duration was extended to 7 days to assess the antiviral effect on the survival rate. Survival time increased to 18–20 days in the MNP/BXM, OSP/BXM, and BXM groups. There was no significant difference in clinical scores compared to the 5-day treatment, except for the MNP/BXM treatment. The clinical scores in MNP/BXM-treated mice were relatively low (2.00 ± 1.73), with most mice showing only neurological signs and dying within 1 day of symptom onset. This may make it difficult to record the clinical scores of dying mice promptly (Table 2). The longer treatment did not improve the survival rate in the OSP and MNP groups. However, survival rates reached 50% in the BXM and two combination treatment groups (*p* < 0.05). Meanwhile, the clinical signs in these groups were also delayed, and death after infection was observed after 14 days (Figure 2(c)).

**Table 2.** Effects of BXM, OSP, and MNP on the survival of mice infected with the bovine H5N1 strain.

Treatment	5-day treatment			7-day treatment		
	Survival time (mean days)	Survival rate (%)	Clinical score* (Mean ± SD)	Survival time (mean days)	Survival rate (%)	Clinical score* (Mean ± SD)
MNP + BXM	12.0	0	5.17 ± 2.04	20.0	50	2.00 ± 1.73
OSP + BXM	11.5	16.7	4.00 ± 2.74	20.5	50	4.33 ± 1.53
MNP	7.0	16.7	4.40 ± 2.19	6.5	0	5.33 ± 1.63
OSP	6.5	0	4.00 ± 2.19	6.0	0	6.00 ± 0.00
BXM	12.0	16.7	5.20 ± 1.79	18.0	50	5.33 ± 1.16
Placebo	5.5	0	6.00 ± 0.00	6.0	0	5.17 ± 2.04

*Clinical score: Only the clinical scores from mice that died or reached the humane endpoint were calculated to reflect the correlation between symptom severity and mortality. The clinical scores of surviving mice were all below 3.

**Figure 3.**
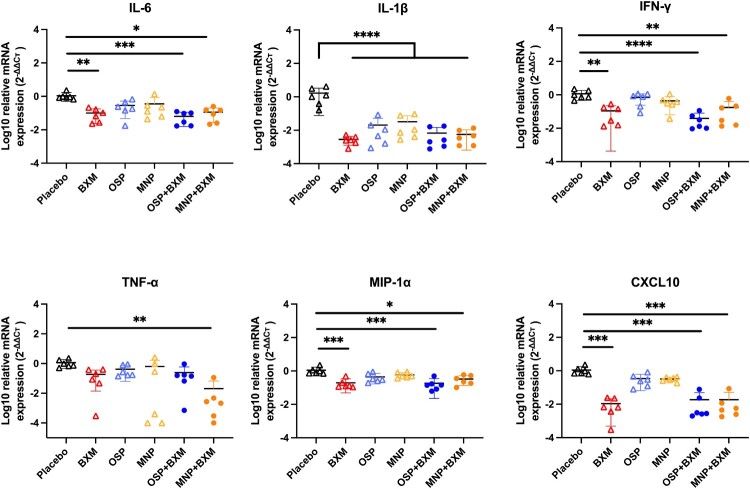
Effect of antiviral treatment on the immune response to the bovine H5N1 virus infection in mice. The inflammatory response in the right lungs of mice at 4 dpi (n = 6). Gene expression was normalized to β-actin and calculated using the 2^-ΔΔct^ method. The results are shown as means ± SD. Statistical analysis was performed using One-way ANOVA. *p < 0.05, **p < 0.01, *** *p* < 0.001.

**Figure 4.**
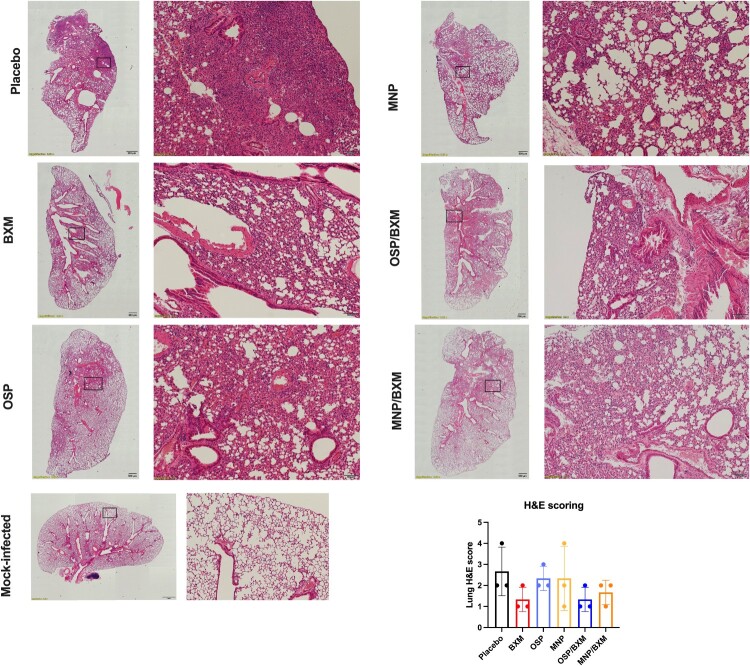
Effect of antiviral treatment on histopathological changes in the bovine H5N1 virus infection. Left lung tissue harvested from the mice (3 mice per group) at 4 dpi was stained with H&E. The images on the left show lung tissue from each group, highlighting pathological changes, with a scale bar of 500μm. The images on the right were magnified sections from the corresponding squares on the left, with a scale bar of 100 μm. The histological score was shown as means ± SD. Statistical analysis was performed using One-way ANOVA.

**Figure 5.**
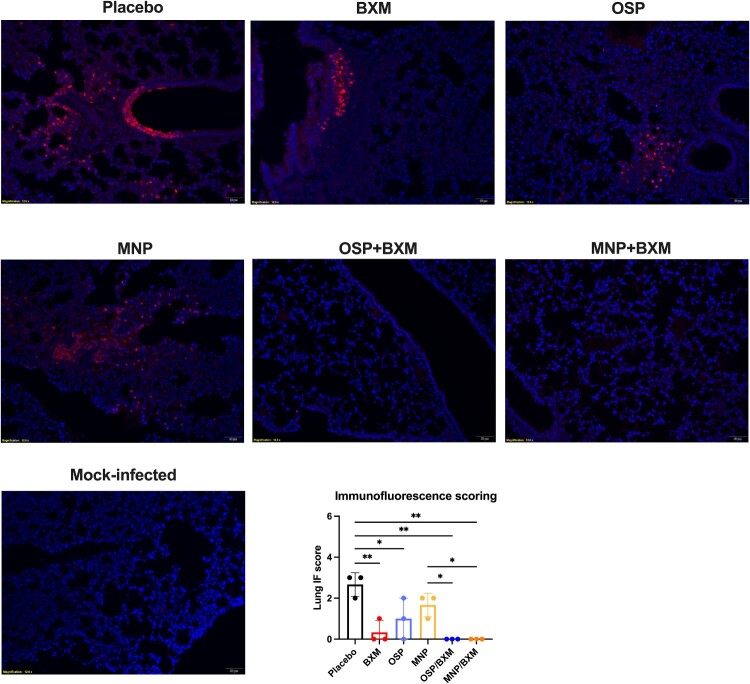
Immunofluorescence results of antiviral treatment against H5N1 virus infection in the lungs of mice. The bovine H5N1 virus infection in the lungs of mice at 4 dpi. The left lung tissue was stained with immunofluorescence (3 mice per group). Rabbit anti-influenza A nucleoprotein (HL1089) and goat anti-rabbit IgG (Alexa Fluor 594) were used. The nuclei were stained with 4′,6-diamidino-2-phenylindole (DAPI). The immunofluorescence score was shown as means ± SD. Statistical analysis was performed using One-way ANOVA.

**Figure 6.**
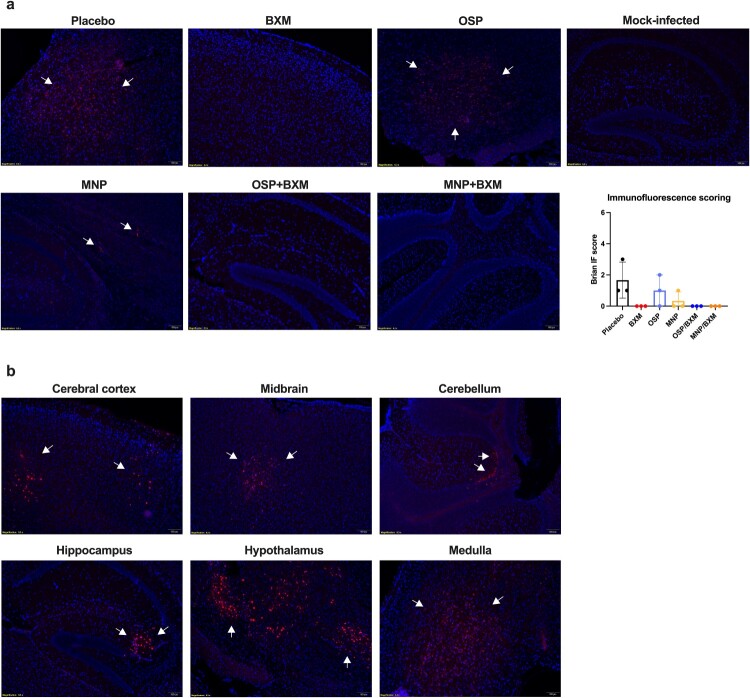
Immunofluorescence results of antiviral treatment against H5N1 virus infection in the brains of mice. The bovine H5N1 virus infection in the left brain of the mouse at 4 dpi (3 mice per group). (a) Effects of monotherapies and combination therapies. The immunofluorescence score was shown as means ± SD. Statistical analysis was performed using One-way ANOVA. (b) The areas of brain infection in the untreated group. Brain tissue was stained with immunofluorescence using rabbit anti-influenza A nucleoprotein (HL1089) and goat anti-rabbit IgG (Alexa Fluor 594), respectively. White arrows indicate the locations of the detected H5N1 virus.

In the untreated group, mice began to show obvious neurological signs and rapid weight loss at 5 dpi. Lung and brain tissues were collected at 4 dpi to assess viral replication and cytokine expression. The results showed that BXM, MNP/BXM, and OSP/BXM treatments significantly reduce viral RNA levels in both the lung and brain (*p* < 0.05). Additionally, MNP and OSP treatments showed an inhibitory trend compared to the placebo, but only OSP significantly decreased viral RNA in the lung (*p* < 0.05) (Figure 2(d)). The combination treatment groups had the lowest viral titers in both tissues (*p* < 0.05). BXM also significantly lowered viral titers in the brain to a minimum, but its inhibitory effect in the lung was weaker than that of the combination therapies, although there was no significant difference (*p* > 0.05). MNP and OSP treatments showed similar antiviral effects in the lungs, reducing viral titers by nearly one log10 compared to the placebo. MNP demonstrated a more effective inhibitory effect than OSP, with only 2 mice showing significant viral replication in their brains, and the average virus titer decreased by approximately 2 log10 compared to the placebo. The viral titer in the OSP group decreased by only one log10, and the virus was detected in half of the mice (Figure 2(e)). Cytokine levels were measured in the lungs of mice. Compared to the placebo group, all antiviral treatment groups showed reduced induction of inflammatory cytokines, especially IL-1β (*p* < 0.05). BXM and the two combination treatments also significantly inhibited the expression of IL-6, IFN-γ, MIP-1α, and CXCL10 (*p* < 0.05). However, only the MNP/BXM combination treatment significantly reduced TNF-α expression (*p* < 0.05) (Figure 3). The histological results showed milder lung inflammation and damage in the MNP/BXM, OSP/BXM, and BXM treatment groups. The placebo group exhibited the most severe lung inflammation and damage, with inflammatory cell infiltration and exudation. However, the scoring results showed no significant difference (Figure 4). Immunofluorescence results in the lungs of mice revealed that viral NP antigen was detected in both the bronchi and alveoli, with the highest detection in the mock-treated group. Monotherapies provided partial inhibition of H5N1 viral replication, while the two combination treatments suppressed viral replication to a minimum at 4 dpi (Figure 5). Viral replication in the brains of mice was also observed in the MNP, OSP, and placebo groups. Most infectious viruses were detected in the placebo group, and the MNP group had fewer viruses than the OSP group. No brain infection was detected in the other three groups (Figure 6(a)). In the placebo group, viral replication was observed in multiple brain regions, including the cerebral cortex, hippocampus, hypothalamus, midbrain, cerebellum, and medulla (Figure 6(b)).

## Discussion

Bovine H5N1 influenza viruses have been detected in the U.S. and have caused human infections. Several studies have demonstrated the antiviral activity and effectiveness of various antiviral drugs against bovine H5N1 virus, including baloxavir, favipiravir, and NAIs, in both *in vitro* and animal models [27-31]. We tested multiple potential antiviral drugs against H5N1 strains isolated from humans and dairy cows *in vitro*. The dual-drug combinations were further tested against A/Thailand/MK2/2004 and A/dairy cow/Texas/24-008749-003/2024 strains, which showed lower susceptibility to OSC. The NHC/BXA and OSC/BXA combinations exhibited significantly greater antiviral effects than monotherapies *in vitro*. Consequently, the prodrugs associated with these two combinations were also tested in a mouse model. The results indicated that BXM played a major role in the antiviral activity, although MNP and OSP also partially inhibited viral replication.

The antiviral effects of these drugs were initially tested against five H5N1 strains. According to the cell viability results, BXA had the lowest EC_50_ value across all 5 strains. Although the EC_50_ of OSC was not lower than that of BXA, OSC exhibited lower cytotoxicity, resulting in a higher SI. However, the EC_50_ of OSC against A/Thailand/MK2/2004 and A/dairy cow/Texas/24-008749-003/2024 H5N1 strains was higher. The IC_50_ of OSC against A/dairy cow/Texas/24-008749-003/2024 H5N1 strain was also higher than that of other strains, consistent with a previous study [30]. On the other hand, the IC_50_ against A/Thailand/MK2/2004 H5N1 strain was similar to that of the other strains. The differences in EC_50_ and IC_50_ for A/Thailand/MK2/2004 H5N1 strain may be due to varying measurement methods. The NA inhibition assay directly assesses sensitivity to NAIs, whereas in the cell viability assay, results are indirectly affected by other factors, such as HA and its interaction with NA. The EC_50_ of RBV was consistent across all 5 strains but was 100-fold and 1000-fold higher than that of OSC and BXA, respectively. The EC_50_ of NHC also remained consistent across all strains. It is the active form of MNP, which inhibits many RNA viruses by incorporating nucleoside analogs into viral RNA [25]. In combination treatments, drug concentrations were set below the EC_50_ to better observe synergistic effects. The results showed that combinations of NHC/BXA and OSC/BXA significantly inhibited viral replication compared to monotherapies. The antiviral effects were tested only in MDCK cells, which differ from human respiratory tract cells. Different ratios of α2,3 – and α2,6-sialoglycans may result in varying degrees of viral infection [32]. It also lacks the structures of the human respiratory tract. Although animal experiments were subsequently conducted, clinical trials are still needed to evaluate treatment in humans.

In mice, lung and brain tissues were collected at 4 dpi to evaluate viral replication in each treatment group. In the placebo group, the mice experienced body weight loss from 3 to 4 dpi, developed severe neurological symptoms, and reached a humane endpoint or died from 5 dpi. The results showed that BXM and the two combination treatments significantly reduced infections in the brains and lungs, especially since the combination treatments maintained viral replication at the lowest levels. However, these combination treatments did not show higher survival rates than BXM alone. Initially, a 5-day treatment was tested, and the results showed that mice on monotherapies died around 7 dpi. In contrast, the BXM and combination treatments extended survival to 9 to 13 dpi but did not prevent death before 14 dpi. In our study, mice showed clinical signs starting from 3-4 dpi, worsening to severe between 5 and 6 dpi. The 5-day treatment, which started at 1 dpi, might not cover later stages of the infection, allowing the virus and the cytokine storm to continue attacking the body and cause tissue damage. As a result, extending the treatment to 7 days increased the 21-day survival rate to 50% in both the BXM and combination groups. Although the survival rate improved, none of the treatments completely prevented death. Viral replication in the lungs was higher than in the brain at 4 dpi, but most mice either died or reached a humane endpoint due to severe neurological signs. Therefore, death might be more directly related to brain infection. The 7-day treatment greatly reduced viral replication, with infectious virus in the lungs and brains of mice receiving the combination therapies becoming almost undetectable. However, 1 MLD_50_ of the bovine H5N1 strain was approximately 1 pfu, meaning that if the virus was not completely eliminated, residual virus could still cause death in the mice. Eisfeld’s study [10] compared the hemagglutination inhibition (HI) titers in ferrets infected with bovine H5N1 and H1N1 viruses at 21 dpi. The results showed that the HI titer of bovine H5N1 virus was lower than that of H1N1 virus. Although it remains unclear whether a lower antibody titer was more influenced by the time of collection or disease severity, it may be associated with a higher risk of death. MNP and OSP did not improve survival rates when used in combination therapies compared to BXM monotherapy. BXM showed a more potent antiviral effect in mice. The OSP and MNP, which had weaker activities, might be overlooked unless an apparent additive or synergistic effect of the dual drug treatment was observed. The result was similar to a previous study of combination therapy against H1N1 virus; the BXM/OSP combination did not produce better clinical outcomes than OSP monotherapy [33]. Nevertheless, compared with BXM treatment, the combination therapies more effectively inhibited viral replication in the lungs of mice. Increasing the concentration of the combination therapy within a safe range may achieve a greater antiviral effect, especially in inhibiting brain infection, thereby increasing survival. In our study, cytokine expression was lower in all treatment groups than in the placebo group, especially in BXM and combination treatments. Cytokine levels are upregulated during infection and may be associated with severe disease [34]. Combination therapy could more effectively inhibit cytokine expression and potentially reduce ICU admissions and mortality in clinical practice. Therefore, combination therapies should be considered for the treatment of bovine H5N1 infection. Some studies have reported that the bovine H5N1 virus isolated from an infected patient caused more severe symptoms and was fatal in mouse and ferret models compared to human infection [28,35]. The milder human infection may make the treatment more effective. However, the 5-day BXM-based therapies might not be sufficient to fully suppress human H5N1 virus infection. The treatment duration should be extended to 7 days, with 80 mg BXM administered once every three days or on alternate days [33]. The use of other drugs in combination therapy should also be prolonged.

Currently, four studies have reported the efficacy of BXM against the bovine H5N1 virus in a mouse model [27-29,31]. The results showed that BXM was effective against H5N1 virus infection, but its efficiency differed from ours. Kiso’s study [29] found that BXM can prevent 100% mortality in mice if treatment begins at 1-hour post-infection (hpi), with the survival rate decreasing to 40% if treatment starts at 1 dpi. The viral dose used in this study was the same as ours, but the BXM dosage was higher, at 50 mg/kg every 12 h for 5 days. Compared to exactly starting treatment at 1 dpi, this study showed a higher survival rate (40% *vs* 16.7%), suggesting that a higher BXM dose yields better antiviral effects. However, this high-concentration treatment may pose safety risks when scaled to human doses. Meanwhile, the immediate treatment after a potential or actual exposure is also challenging in real-world settings. The dosage and timing of treatment initiation used in Gu's study also differed from ours [28]. Jones’s study [27] showed significant antiviral effects of BXA and OSP. The survival rates for high-dose (25 mg/kg) and low-dose (5 mg/kg) BXA were 75% and 50%, respectively. The high-dose OSP (200 mg/kg every 12 h) protected about 35% to 40% of the mice from death. The inoculation dose administered intranasally in this study was reduced to 5 MLD_50_, and BXA instead of BXM was given subcutaneously. Pascua’s study [31] showed that 15 mg/kg BXM completely protected mice from lethality, but the inoculation dose used in this study was 5 MLD_50,_ and the treatment started at 2 hpi. Therefore, the antiviral effects could not be directly compared because of differences in infection and treatment conditions.

One limitation of our study is that we did not test different drug doses *in vivo*. The drug concentrations used in mice were calculated based on clinical dosing, frequency, and route of administration. In mice, 10 mg/kg of OSP twice daily for 5 days was considered equivalent to the human dose [36]. The BXM dose was based on previous studies, which demonstrated that a concentration of 15 mg/kg every 12 h was comparable to the human single dose [37,38]. However, 7-day treatment with fixed drug concentrations significantly improved the survival rate of mice, and this extended treatment for three drugs was also shown to be safe [39-41]. Additionally, we did not evaluate different treatment timings; all drugs were administered starting from 1 dpi. Early treatment provided stronger antiviral protection [29,31], but it may be challenging to implement in clinical practice due to limited medical resources and a lack of awareness about protection among exposed individuals. Therefore, our results are more applicable to clinical medication.

In summary, BXA, NHC, OSC, and RBV can significantly inhibit the replication of various H5N1 viruses *in vitro*. The combination treatments of NHC/BXA and OSC/BXA showed stronger antiviral effects. In mice, the 7-day BXM, MNP/BXM, and OSP/BXM treatments significantly increased survival rates. Combination treatments significantly suppressed the viral replication in both the lungs and brains of mice. Therefore, extended BXM-based combination therapies could be considered as a first-line treatment for humans. The results provided a reference for clinical treatment.

## Supplementary Material

Supplementary Figure 1.tiff

R4_Supplementary_Methods-clean.docx

Supplementary Figure 2.tiff

R4 clean manuscript.docx

Supplementary Tables.docx

## Data Availability

The data supporting the study's findings are available from the corresponding authors upon reasonable request**.**
