# Supplementary figures and images for: Antiviral activities of multiple antivirals against highly pathogenic avian influenza A H5N1 in vitro and in mice
Source: Emerg Microbes Infect. 2026 Mar 31;15(1):2645843. doi: 10.1080/22221751.2026.2645843 (PMC13040577; doi:10.1080/22221751.2026.2645843)

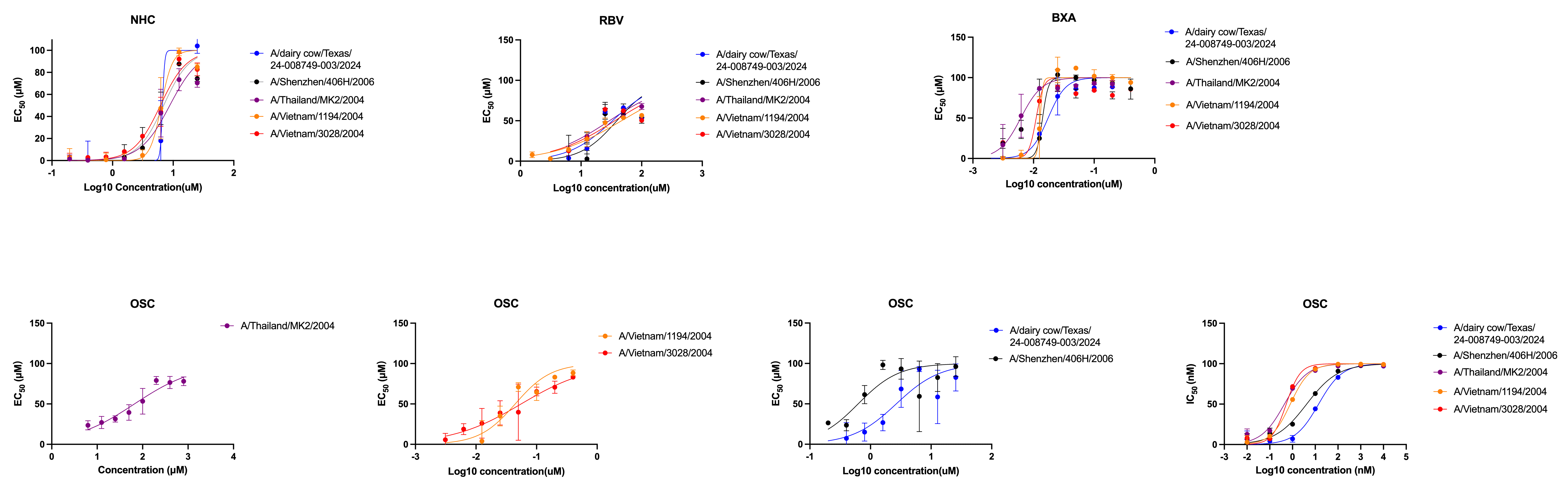

Supplement: Supplementary Figure 1.tiff [file TEMI_A_2645843_SM4553.tiff]
