# Supplementary material for: Antiviral activities of multiple antivirals against highly pathogenic avian influenza A H5N1 in vitro and in mice
Source: Emerg Microbes Infect. 2026 Mar 31;15(1):2645843. doi: 10.1080/22221751.2026.2645843 (PMC13040577; doi:10.1080/22221751.2026.2645843)
Supplement: R4_Supplementary_Methods-clean.docx [file TEMI_A_2645843_SM4552.docx]

**Supplementary Methods**

***Viruses, cell lines, and compounds***

The H5N1 viruses including A/Thailand/MK2/2004 (Clade 1), A/Vietnam 1194/2004 (Clade 1, NCBI: txid644788), A/Vietnam/3028/2004 (Clade 1, NCBI: txid261268), A/Shenzhen/406H/2006 (Clade 2.3.4, GenBank accession No. EF137706–EF137713) and A/dairy cattle/Texas/24-008749-003/2024 (Clade 2.3.4.4b, GISAID No. EPI_ISL_19014386) were studied. Whole-genome sequencing was completed for all viruses, and no significant mutations associated with drug resistance were found using GISAID FluSurver. All strains were isolated from humans, except A/dairy cow/Texas/24-008749-003/2024. which was rescued using reverse genetics [1,2]. All virus stocks were prepared and titrated using plaque assays on Madin-Darby canine kidney (MDCK) cells. The compounds used in our study were purchased from MedChemExpress (USA). Oseltamivir carboxylate (OSC) and EIDD-1931 (NHC) were dissolved in water, while baloxavir acid (BXA) and ribavirin (RBV) were dissolved in dimethyl sulfoxide (DMSO) for *in vitro* studies. In the animal study, oseltamivir phosphate (OSP) was dissolved in water, whereas molnupiravir (MNP) and baloxavir marboxil (BXM) were dissolved in 50% polyethylene glycol 300 (PEG300) in water. All compounds were stored at -80℃.

***Antiviral treatments in vitro***

The antiviral treatments were applied to MDCK cells. First, the cytotoxicity concentrations were determined. The highest working concentrations of the four drugs were 50-fold dilutions of their stock solutions: BXA at 200 μM, OSC at 2000 μM, NHC at 1000 μM, and RBV at 2000 μM. All drugs were serially diluted 4-fold in fresh Eagle's minimal essential medium (MEM), and added to the cells, and cell viability was tested at 72 hours. Cell viability was determined using the Thiazolyl Blue Tetrazolium Bromide (MTT) assay. The supernatant was discarded, and 100 μl of 0.5mg/ml MTT was added to each well, which was incubated at 37℃ in the dark for 3h. Then 100 μl of 0.01M SDS-HCI was added to dissolve the precipitated formazan crystals. The experiment included a DMSO control, an untreated (MEM) control, and a blank control. The highest DMSO concentration used was 2%, which showed no obvious cell toxicity. Absorbance was measured at 560 nm, and cell viability was calculated as OD (treated - blank) / OD (untreated - blank). The 50% cytotoxic concentration (CC_50_) was calculated from cell viability data. Treatments were set up in triplicate, and the experiments were repeated twice.

*In vitro* antiviral treatments showed that all drug concentrations had no apparent toxicity, with the highest concentrations maintaining cell viability above 80%. For monotherapies, five H5N1 strains were diluted to 0.0005 MOI for cell inoculation. After 1 hour of incubation at 37℃, the viruses were replaced with 2-fold serially diluted drugs in fresh MEM without TPCK-trypsin. The concentrations of NHC ranged from 0.20 μM to 25 μM, RBV from 3.125 μM to 100 μM, and BXA from 0.002 μM to 0.4 μM. The total concentrations of OSC ranged from 0.003 μM to 800 μM, and appropriate serial dilution ranges were selected for each H5N1 strain. The experiments also included untreated controls and uninfected cell controls. Because the highest concentrations of BXA and RBV contained less than 0.1% DMSO, the uninfected cell control was treated with only fresh MEM. After 72 hours, cell viability was assessed using the MTT assay, and supernatants were collected for virus titration using TCID_50_. The 72-hour time point was chosen because viral replication was near its peak, as observed from the pilot experiment and previous studies [3]. Cell viability was calculated as OD (treated - untreated) / OD (uninfected - untreated). The 50% effective concentration (EC_50_) was calculated from cell viability data. Treatments were set up in triplicate, and the entire experiment was repeated twice. The antiviral efficacy of OSC against all strains was also evaluated using the NA inhibition assay, which employed the NA-Fluor influenza neuraminidase assay kit (Applied Biosystems, USA), according to the manufacturer’s instructions. The 50% inhibitory concentration (IC_50_) was calculated from the fluorescence readings with treatments set up in duplicate and experiments repeated twice. Based on cell viability results, supernatants from concentrations near the EC_50_ were used for virus titration via TCID_50_. The culture medium used was MEM without TPCK-trypsin.

The A/Thailand/MK2/2004 and A/dairy cow/Texas/24-008749-003/2024 H5N1 strains were selected for evaluation of *in vitro* combination treatments. Dual drug treatment involved BXA combined with NHC, OSC, or RBV. The concentration of BXA was fixed at 0.005 μM, while NHC ranged from 3.125 μM to 25 μM, RBV ranged from 1.56 μM to 100 μM, and OSC ranged from 3.125 μM to 200 μM. The experiments also included untreated controls and uninfected cell controls. In both monotherapy and combination therapy, supernatants were collected at 72 hours, and viral titers were determined using the TCID_50_ method. All treatments were set up in triplicate, and the experiments were repeated twice.

***Animals***

Female BALB/c mice, aged 6-8 weeks, were purchased from the Centre for Comparative Medicine Research (CCMR) at HKU. The mice were transferred to a biosafety level 3 animal laboratory 5‐7 days before the experiments. After virus inoculation, the mice were monitored daily for clinical signs and body weight changes. Any animal losing more than 20% of its body weight or showing signs of severe disease was euthanized. The evaluation criteria were shown in Supplementary Table 1. The study was approved by the Committee on the Use of Live Animals in Teaching and Research at the University of Hong Kong (CULATR No. 24-171).

***Virus inoculation of mice***

Mice were intranasally inoculated with the recombinant H5N1 strain A/dairy cow/Texas/24-008749-003/2024, with a total volume of 20 μl after being anesthetized with ketamine (100 mg/kg) and xylazine (10 mg/kg). The 50% mouse lethal dose (MLD_50_) was first determined, and the virus dose was set as a 10-fold serial dilution ranging from 1 pfu to 1000 pfu. A non-infected control group was also included. Each group consisted of 4 mice. The body weight changes and survival rates were monitored and recorded for each group, with the longest observation period lasting 14 days post-infection (dpi). In the antiviral treatment experiments, mice were inoculated with 10 pfu (approximately 10 times the 50% mouse lethal dose, 10 MLD_50_) of the virus to establish a moderate-to-severe infection model.

***Antiviral treatments in mice***

The antivirals used in mice include BXM, OSP, and MNP. The concentrations and frequencies of OSP and MNP used in mice were derived from clinical practice for influenza and SARS-CoV-2 viruses, respectively. The human dose is multiplied by 12.3 to obtain the mouse equivalent dose based on body surface area [4]. These two drugs were administered orally twice daily at concentrations of 10 mg/kg for OSP and 125 mg/kg for MNP. The treatments started from 1 dpi, and mice were treated for 5 or 7 days. BXM was administered orally at a dose of 15 mg/kg twice daily, following the method described in previous studies, which showed that this dose and frequency yielded plasma concentrations similar to a single human dose [5]. Each drug in the combination therapy was administered in the same way as in the monotherapy. Six groups were established: MNP/BXM and OSP/BXM combination treatment groups, as well as MNP, OSP, BXM, and placebo treatment groups. All the treatments were started at 1 dpi. The efficacy of 5-day and 7-day treatments was evaluated separately. Lung and brain tissues from the mice were collected at 4 dpi for virologic, histopathologic, and immunologic analyses. This time point was chosen based on the survival duration of mice in the placebo group. Clinical signs, body weight changes, and survival rates were monitored until 14 dpi in the 5-day treatment group and 21 dpi in the 7-day treatment group. Mice that developed severe infections and reached humane endpoints were considered dead. The two time points, 4 dpi and either 14 or 21 dpi, were set up and assigned to each treatment group with a sample size of 6 mice per group at each time point. The sample size was determined using the Power & Animal number calculator provided by the CCMR at HKU.

***Virologic and immunologic analysis of mouse tissues***

The right lung and right brain tissues of mice (6 mice per group) were collected for virologic analysis. Tissues were immersed in 1 ml MEM with 1% penicillin/streptomycin after sampling, and bead-homogenized using a TissueLyser II (Qiagen) at 28Hz oscillation frequency for 2 min twice. The homogenate was clarified by centrifugation and stored at −80°C. The virus titer was determined by a plaque assay. MDCK cells were seeded in 24-well plates one day before infection and incubated at 37℃_._ Plates with a monolayer of cells covering the bottom of the wells were used for the assay. The cells were washed twice with 1x phosphate-buffered saline (1x PBS) before the inoculation. Then, the serially diluted virus was added to the cells and incubated at 37 ℃ for 1 hour. After incubation, the plates were washed twice with fresh MEM. The 2% agarose was heated to 80℃ to liquefy and then mixed in equal parts with 2x MEM without TPCK-trypsin. When the agarose cooled to about 37 ℃, the MEM on the cells was discarded and replaced with the agarose. The plates were flipped 180^o^ after the agarose solidified and incubated at 37℃. After 72 hours, 10% PBS-buffered formaldehyde was added to the wells overnight for cell fixation. Finally, the formaldehyde and semi-solid agarose were removed, and crystal violet was added for cell staining. Cell counting was performed to determine the virus titer. The limit of detection (LOD) of the plaque assay was 20 pfu/ml. RT-qPCR was used to determine the viral load and cytokine expression. Total RNA was extracted using the RNeasy Mini Kit (Qiagen), and reverse transcription was performed using PrimeScript RT‐Master Mix lit (TaKaRa). RT-qPCR was performed on the StepOne Real‐Time PCR system (Applied Biosystems) with the SYBR Premix Ex Taq II kit (TaKaRa). Primers targeted the influenza A M gene and cytokines, including IL-6, IL-1β, IFN-γ, TNF-α, MIP-1α, and CXCL10 [6-8]. The β-actin gene served as a housekeeping gene for normalization, and relative quantification was calculated using the 2^‐ΔΔct^ method. The primer sequences are listed in Supplementary Table 2. The LOD of qPCR for the influenza A M gene was 0.1 TCID_50_ per ml.

***Histopathology and immunofluorescence staining of mouse tissues***

The left lung and left brain tissues (3 mice per group) were fixed in 10% formalin and embedded in paraffin. Five mm tissue sections were stained with hematoxylin and eosin (H&E) and by immunofluorescence. A semi-quantitative scoring system was used to assess lung lesions. Damage was assessed based on infiltration and exudation in the peribronchiolar, perivascular, and alveolar spaces, as well as luminal cell detachment. Scores were 0 = no lesion, 1 = lesion covering up to 5%, 2 = up to 25%, 3 = up to 50%, 4 = more than 50%. For immunofluorescence, the primary antibody was rabbit anti-influenza A nucleoprotein (HL1089), and the secondary antibody was goat anti‐rabbit IgG (Alexa Fluor 594). Nuclei were stained with 4′,6‐diamidino‐2‐phenylindole (DAPI). The images were captured with an Olympus BX53F semimotorized fluorescence microscope using Olympus cellSens Dimension Imaging software. A scoring system was also used to assess the area of viral infections in the lungs and brains of mice. The score level was the same as the pathological scoring.

**Reference**

1. Hoffmann E, Neumann G, Kawaoka Y, et al. A DNA transfection system for generation of influenza A virus from eight plasmids. Proc Natl Acad Sci U S A. 2000 May 23;97(11):6108-13.

2. Song W, Wang P, Mok BW, et al. The K526R substitution in viral protein PB2 enhances the effects of E627K on influenza virus replication. Nat Commun. 2014 Nov 20;5:5509.

3. Li IW, Chan KH, To KW, et al. Differential susceptibility of different cell lines to swine-origin influenza A H1N1, seasonal human influenza A H1N1, and avian influenza A H5N1 viruses. J Clin Virol. 2009 Dec;46(4):325-30.

4. Nair AB, Jacob S. A simple practice guide for dose conversion between animals and human. J Basic Clin Pharm. 2016 Mar;7(2):27-31.

5. Fukao K, Noshi T, Yamamoto A, et al. Combination treatment with the cap-dependent endonuclease inhibitor baloxavir marboxil and a neuraminidase inhibitor in a mouse model of influenza A virus infection. J Antimicrob Chemother. 2019 Mar 1;74(3):654-662.

6. Zhang AJ, Li C, To KK, et al. Toll-like receptor 7 agonist imiquimod in combination with influenza vaccine expedites and augments humoral immune responses against influenza A(H1N1)pdm09 virus infection in BALB/c mice. Clin Vaccine Immunol. 2014 Apr;21(4):570-9.

7. Devarapu SK, Grill JF, Xie J, et al. Tumor necrosis factor superfamily ligand mRNA expression profiles differ between humans and mice during homeostasis and between various murine kidney injuries. J Biomed Sci. 2017 Sep 19;24(1):77.

8. Lai JH, Wu DW, Huang CY, et al. Induction of LY6E regulates interleukin-1β production, potentially contributing to the immunopathogenesis of systemic lupus erythematosus. Cell Commun Signal. 2025 Mar 20;23(1):146.
