# Supplementary material for: Antiviral activities of multiple antivirals against highly pathogenic avian influenza A H5N1 in vitro and in mice
Source: Emerg Microbes Infect. 2026 Mar 31;15(1):2645843. doi: 10.1080/22221751.2026.2645843 (PMC13040577; doi:10.1080/22221751.2026.2645843)
Supplement: Supplementary Tables.docx [file TEMI_A_2645843_SM4549.docx]

**Supplementary Table 1. Humane endpoint scoring**

| Score | 0 | 1 | 2 | 3 |
| --- | --- | --- | --- | --- |
| Activity | normal | isolated | Huddled/inactive | Fitting, unresponsive |
| Posture | normal | asymmetric posture, mild hunching | persistent/severe hunching | trembling |
| Movement | normal | slight incoordination | tiptoe walking or reluctance to move | staggering/limb dragging/paralysis/seizure, etc. |
| Coat condition | normal | rough | ruffled/unkempt | bleeding or infected wounds or self-mutilation |
| Breathing | normal | rapid/shallow | rapid abdominal breathing | labored, irregular, blue skin |

Criteria for euthanasia: body weight loss >20% or signs of severe disease (score ≥ 3).

**Supplementary Table 2. List of primer sequence**

| **Gene name** | **Forward primer (5’ to 3’)** | **Reverse Primer (5’ to 3’)** |
| --- | --- | --- |
| *FluA M* | GACCRATCCTGTCACCTCTGAC | AGGGCATTYTGGACAAAKCGTCTA |
| *β-actin [1]* | ACGGCCAGGTCATCACTATTG | CAAGAAGGAAGGCTGGAAAAG |
| *IL-1β [1]* | GCCTTGGGCCTCAAAGGAAAGAATC | GGAAGACACAGATTCCATGGTGAAG |
| *IL-6 [1]* | TGGAGTCACAGAAGGAGTGGCTAAG | TCTGACCACAGTGAGGAATGTCCAC |
| *TNF-α [2]* | GGTGCCTATGTCTCAGCCTCTT | GCCATAGAACTGATGAGAGGGAG |
| *IFN-γ [1]* | AAGCGTCATTGAATCACACC | CGAATCAGCAGCGACTCCTT |
| *MIP-1α [1]* | CCCAGCCAGGTGTCATTTTCC | GCATTCAGTTCCAGGTCAGTG |
| *CXCL10 [3]* | ATCATCCCTGCGAGCCTATCCT | GACCTTTTTTGGCTAAACGCTTTC |

**Reference**

1. Zhang AJ, Li C, To KK, et al. Toll-like receptor 7 agonist imiquimod in combination with influenza vaccine expedites and augments humoral immune responses against influenza A(H1N1)pdm09 virus infection in BALB/c mice. Clin Vaccine Immunol. 2014 Apr;21(4):570-9.

2. Devarapu SK, Grill JF, Xie J, et al. Tumor necrosis factor superfamily ligand mRNA expression profiles differ between humans and mice during homeostasis and between various murine kidney injuries. J Biomed Sci. 2017 Sep 19;24(1):77.

3. Lai JH, Wu DW, Huang CY, et al. Induction of LY6E regulates interleukin-1β production, potentially contributing to the immunopathogenesis of systemic lupus erythematosus. Cell Commun Signal. 2025 Mar 20;23(1):146.
